# Effects of political versus expert messaging on vaccination intentions of Trump voters

**DOI:** 10.1371/journal.pone.0257988

**Published:** 2021-09-27

**Authors:** Christopher T. Robertson, Keith Bentele, Beth Meyerson, Alex S. A. Wood, Jackie Salwa

**Affiliations:** 1 School of Law, Boston University, Boston, Massachusetts, United States of America; 2 College of Social and Behavioral Sciences, University of Arizona, Tucson, Arizona, United States of America; 3 College of Social and Behavioral Sciences, University of Arizona, Tucson, Arizona, United States of America; 4 University of Arizona, Tucson, Arizona, United States of America; 5 Student, Harvard Law School, Cambridge, Massachusetts, United States of America; University of Haifa, ISRAEL

## Abstract

To increase COVID-19 vaccine uptake in resistant populations, such as Republicans, focus groups suggest that it is best to de-politicize the issue by sharing five facts from a public health expert. Yet polls suggest that Trump voters trust former President Donald Trump for medical advice more than they trust experts. We conducted an online, randomized, national experiment among 387 non-vaccinated Trump voters, using two brief audiovisual artifacts from Spring 2021, either facts delivered by an expert versus political claims delivered by President Trump. Relative to the control group, Trump voters who viewed the video of Trump endorsing the vaccine were 85% more likely to answer “yes” as opposed to “no” in their intention to get fully vaccinated (RRR = 1.85, 95% CI 1.01 to 3.40; *P* = .048). There were no significant differences between those hearing the public health expert excerpt and the control group (for “yes” relative to “no” RRR = 1.14, 95% CI 0.61 to 2.12; *P* = .68). These findings suggest that a political speaker’s endorsement of the COVID-19 vaccine may increase uptake among those who identify with that speaker. Contrary to highly-publicized focus group findings, our randomized experiment found that an expert’s factually accurate message may not be effectual to increase vaccination intentions.

## Background

Political polarization is a barrier to fighting COVID-19. Several subpopulations are of concern, including the nearly half of Republicans who said in April 2021 that they will decline the vaccine [1]. In addition to their own risk, unvaccinated populations facilitate viral mutations that undermine efficacy of vaccines.

Leading Republican pollster Frank Lutz and Dr. Tom Frieden, a former director of the U.S. Centers for Disease Control and Prevention, conducted a highly-publicized focus group, which found that Americans who identified as Republicans were receptive to five factual claims delivered by an expert, but were not receptive to advice delivered by politicians, including House Minority Leader Kevin McCarthy (Calif.), Sen. Bill Cassidy (La.) and Rep. Brad Wenstrup (Ohio), chair of the GOP Doctors Caucus [2]. Attendees “universally” said they would not be persuaded by hearing from former President Donald Trump either [2]. This approach seeks to de-politicize vaccines so patients can make evidence-based choices. On the other hand, polls suggest that voters who support former President Donald Trump trust him for medical advice more than they trust public health authorities [3].

In fact, Former President Trump endorsed the COVID-19 vaccine in a March 2021 interview on Fox News, claiming credit for it, and disclosing that he and the First Lady Melania Trump had received it [4]. Yet, by this time, Trump no longer had the platforms of the White House or Twitter, which may have reduced the impact of his endorsement.

The relationship between political identities, political leaders, and personal health behaviors is complex and understudied [5]. A 2020 study of 3,000 American adults found that political identities were the strongest predictors of personal health behaviors in the COVID-19 pandemic [6]. A 2021 study suggests that people will respond positively to the leaders of their party that are endorsing the COVID-19 vaccine, but negatively to the leaders of the other major political party [7]. The study exposed people who identified as Republicans to a message from former President Trump, current President Biden, and a neutral control with no endorsements. The study found an increase of 7.0% higher vaccination intentions in unvaccinated Republicans after viewing the Republican endorsement compared to those that viewed the Democratic endorsement. This effect is further highlighted by the observation that Republicans who viewed the Democratic endorsement stated that they would be “significantly less likely to encourage others to vaccinate and had more negative attitudes toward the vaccine”.

Politics are not the only dimension of potential shared identities, however. A 2017 study found that donations among conservatives increase when they feel accountable to a liberal audience with whom they have a “salient shared identity” [8]. Though the study noted that if the political identity is stronger than the salient identity, then this phenomenon is no longer present. Conformity may be a powerful motivator and further research should be done on other factors that may be stronger than political identity.

## Objective

We sought to test the efficacy of factual versus political approaches for effective vaccine communication to determine their effects on vaccination intentions.

## Methods

We conducted an online, randomized experiment on March 23, 2021 with a group of respondents, recruited from Amazon Mechanical Turk, using the CloudResearch platform (formerly known as “TurkPrime”) [9]. To recruit respondents with an offer of $1.55 each, our task was described as “University research! share your views! quick survey.” To ensure quality respondents, we used CloudResearch to block duplicate IP addresses and suspicious geocodes, and verified worker country location and successful passage of Cloud Research quality checks on past HITs. We also required 85% success by workers on prior tasks on the Mturk platform. Respondents spent 8.27 (6.57) minutes on the task at the mean (median).

Our initial sample was set at 500 responses, based on funds available for the opportunistic research project. Given prior surveys showing vaccine resistance in particular populations, we specified eligibility for our experiment to be limited respondents indicating that they voted for Donald J. Trump in the 2020 election or would have done so if they could have voted. Before analyzing outcomes data, we screened to include only those who completed the full survey and who were not flagged by the Qualtrics survey software as “ballot stuffers” (who may have taken the survey a second time after being exposed to a different experimental condition the first time). The foregoing screens reduced our sample to N = 434.

For our final sample for analysis, we also screened to include only those who indicated that they had *not* yet been fully vaccinated. The final sample for analysis is N = 387, with demographics shown in Table 1. The population was relatively young (71% under age 50), likely in part due to the fact that older populations had already received priority for vaccination as of the date of the study. Respondents were also well educated in this convenience sample (60% had BA degrees or more).

**Table 1:** Descriptive statistics (N = 387).

Variable	Proportion of the Sample
**Already Vaccinated**	
No	0.87
One dose	0.13
**Treatment Condition**	
Trump Video	0.37
Frieden Video	0.30
No Video	0.33
**Age**	
18 to 29	0.14
30 to 49	0.57
50 to 64	0.23
> 64	0.06
**Gender**	
Male	0.52
Female	0.48
**Household Income**	
$0 to $29,999	0.18
$30,000 to $59,999	0.31
$60,000 to $149,999	0.42
$150,0000 or more	0.09
**Education Level**	
Less than HSD/HSD/GED	0.13
Some College or Associates Degree	0.28
Bachelors Degree or higher	0.59
**Race/Ethnicity**	
Person of Color	0.17
Spanish, Hispanic, or Latino	0.07
**Political Party Affiliation**	
Republican	0.65
Democrat	0.05
Independent	0.24
None/Other	0.06
**Religious Views**	
Born-again or Evangelical Christian	0.27

We randomly assigned respondents to a control condition or two experimental conditions, shown in Table 2. In one condition, respondents heard an excerpt of Dr. Frieden explaining the five facts delivered in the highly-publicized focus group, while seeing an image of his face and title [2]. In another condition, respondents heard an excerpt from former President Trump endorsing the vaccine on Fox News, while seeing images from that broadcast [4]. The outcome was whether respondents intended to get fully vaccinated (yes, no, or unsure).

**Table 2:** Experimental stimuli.

Condition	Audio Transcript
Frieden Video	“[**Frieden**:] **One**–if you get infected with the virus it will go all over your body and stay there for at least a week and be much more likely to cause you long-term problems than the vaccine. **Two**–if you get the vaccine, it will prime your immune system, but then the vaccine is gone. It will not be with you anymore. **Three**–more than 95% of the doctors who have been offered this vaccine have gotten it as soon as they can. **Four**–the more we vaccinate, the faster we can get back to growing our economy and getting jobs. **Five**—if people get vaccinated, we’re going to save at least 100,000 lives of Americans that would otherwise be killed by COVID.” (53 seconds)
Trump Video	“[**Trump**:] And I’ve always felt it was the most important, the vaccine. The key was always going to be the vaccine. It works incredibly well. 95%, maybe even more than that. It works incredibly well, and it’s really saving our country, and it’s saving frankly the world. [**Interviewer**:] So… so Mr. President I know that you received the vaccine, Mrs. Trump also got the vaccine, would you recommend to our audience that they get the vaccine then? [**Trump**:] I would. I would recommend it, and I would recommend it to a lot of people that don’t want to get it and a lot of those people voted for me frankly, but again you know we have our freedoms and we have to live by that and I agree with that also, but it’s a great vaccine, it’s a safe vaccine, and it’s something that works and uh we’ve been working—[**Interviewer**:] Well- [**Trump**:] round the clock and what I got the FDA to do this would have happened, this would have happened in many, many years from now if I didn’t get involved and if we didn’t get involved. [**Interviewer**:] Yes–” (63 seconds)
Control	[None]

Our project was cleared by the Institutional Review Board at Boston University as exempt, as it concerned routine survey procedures. All respondents provided informed consent by indicating their agreement at the end of a page of terms stating their rights as research participants, clicking “next” to start the survey instrument on the Qualtrics platform. Our data and STATA replication file are posted on the Open Science Framework [10].

## Findings

In the no-video control group, 35% (CI 27% to 44%) responded “no” they would not vaccinate. In the Frieden condition, 34% (CI 26% to 44%) said “no.” In the Trump condition, only 24% (CI 17% to 32%) said “no” they would not vaccinate.

**Fig 1:**
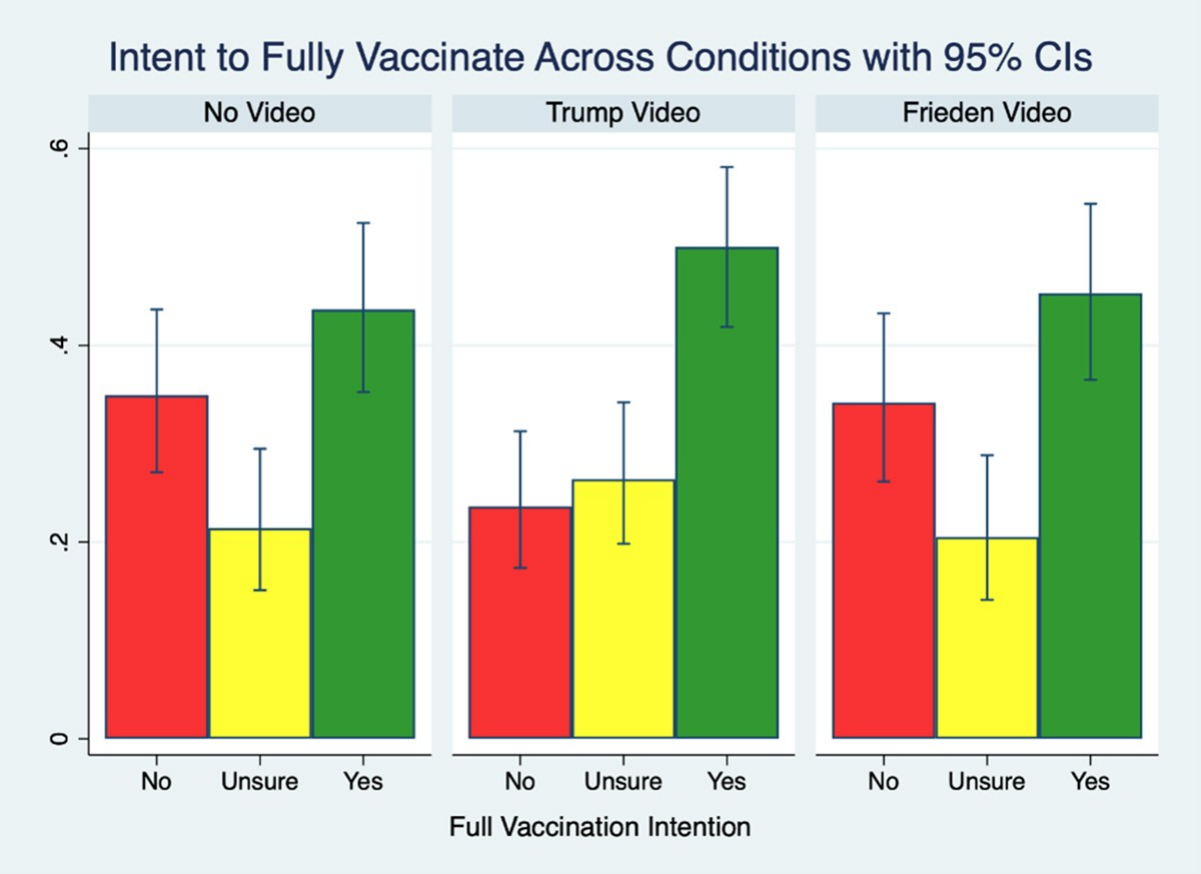
Vaccination intention by experimental condition, with 95% confidence intervals.

**Fig 2:**
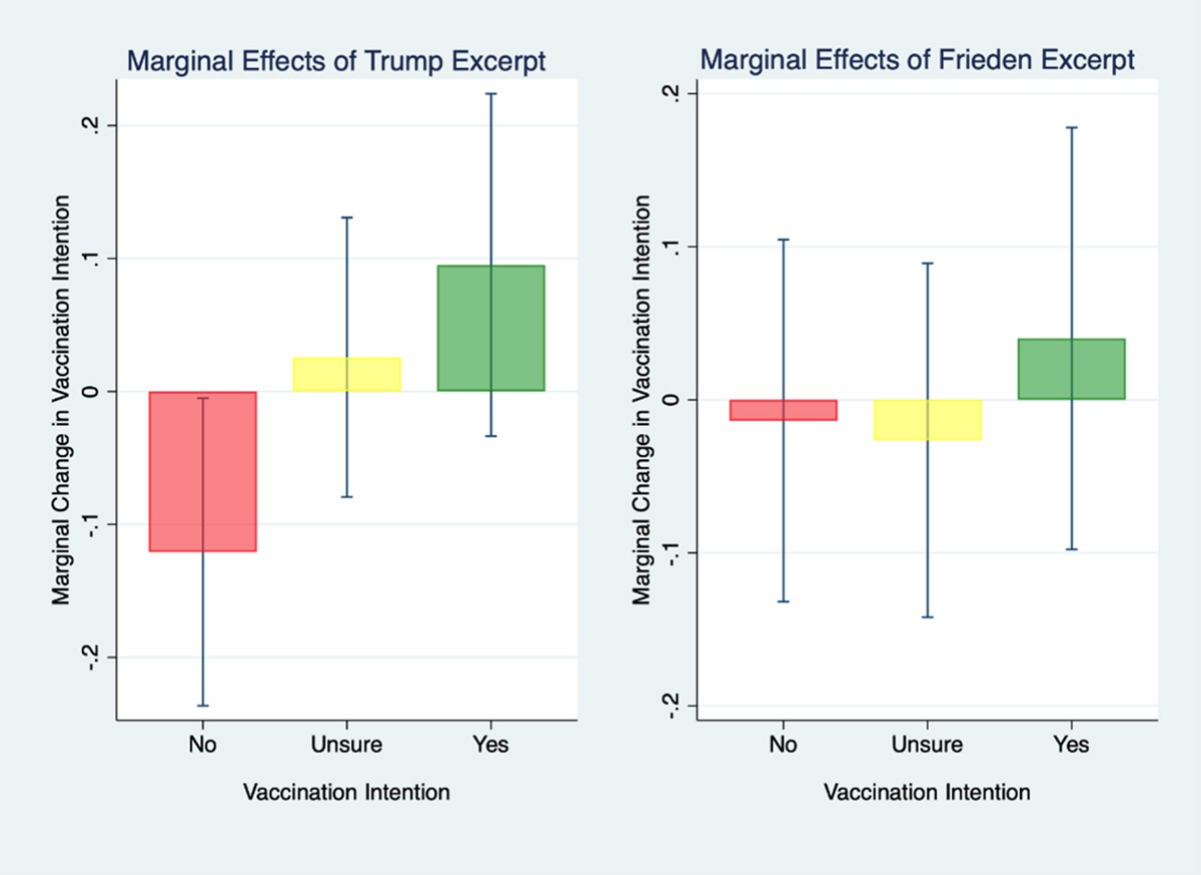
Marginal effects on vaccination intention compared to control group by experimental condition, with 95% confidence intervals.

Given that respondents’ intention to get fully vaccinated is captured with a three-category response option (yes, no, unsure), we use multinomial logistic regression to estimate the odds of respondents answering “yes” relative to “no” and “unsure” relative to “no” while controlling for the demographic and political variables listed in Table 1. Relative to the control group, Trump voters who viewed the video of Trump endorsing the vaccine were 85% more likely to answer “yes” as opposed to “no” in their intention to get fully vaccinated (RRR = 1.85, 95% CI 1.01 to 3.40; *P* = .048). There were no significant differences between those hearing the Frieden excerpt and the control group (for “yes” relative to “no” RRR = 1.14, 95% CI 0.61 to 2.12; *P* = .68). In order to get a more intuitive sense of what these relative odds mean in practice, Fig 1 displays the actual proportions of respondents answering “yes”, “no”, and “unsure” across the different experimental conditions. Fig 2 displays the marginal effect of the Trump excerpt treatment on vaccination intention with all variables set to their means.

Of the 387 individuals included in these analyses, 337 were unvaccinated and 50 had received their first dose. If we drop these 50 partially-vaccinated individuals from the analyses to only focus on “those who did not have the first dose” (as a reviewer requested), then the RRR for the Trump video is unchanged and the p-value increases slightly (RRR = 1.85, 95% CI 0.98 to 3.51; P = .059).

As a secondary outcome, an index variable measured perceptions of vaccine safety and efficacy. This index is the average of three questions: “How safe do you think the COVID-19 vaccines are?”, with a 5-category response option ranging from “extremely safe” to “not at all safe”. “How effective do you think the COVID-19 vaccines are to protect the person receiving it?” and “How effective do you think the COVID-19 vaccines will be to end the pandemic?”. These second two questions also have a 5-category response option ranging from “extremely effective” to “not effective at all”. These three variables were highly correlated, with a Cronbach’s alpha of .94 indicating that this index has a very high level of internal consistency. Using this outcome variable in an Ordinary Least Squares (OLS) regression model with bootstrapped standard errors and the same controls, the Trump excerpt increased the average score of respondents on the 4-point index by .3 points (Coef. = .29, 95% CI .002 to .58; *P* = .048). In contrast, the Frieden excerpt had no significant impact (Coef. = .09, 95% CI -.20 to .38; *P* = .56). We also asked whether respondents viewed COVID-19 a hoax versus a serious problem, and neither excerpt made a difference (Trump excerpt Coef. = 1.99, 95% CI -5.0 to 9.0; *P* = .58; Frieden excerpt Coef. = -.71, 95% CI -8.2 to 6.8; *P* = .85).

## Limitations

This randomized vignette experiment has several limitations. Most notably, we are not observing actual vaccination behaviors, but rather only expressed intentions to get vaccinated. Our study and analyses were not pre-registered, though the primary endpoint and relatively straightforward experimental design reduces the risk of p-hacking.

As applied research, we used complex real-world communication artifacts that combine different messages (one focusing on five key facts, the other expressing pride in having supported the invention of the vaccines) with different speakers (Trump and Frieden). Accordingly, this study design cannot isolate the effects of each component.

We tested for effects on Trump voters; the results are unlikely to be generalizable to others, such as those who voted against Trump. Nonetheless it is feasible for public health communicators to target audiences with particular messages and messengers, given that different populations of Americans tend to consumer different mass media (e.g., Fox News versus MSNBC) and that social media messages can be targeted at the individual-level.

We conducted the study at a particular moment in the COVID-19 pandemic’s trajectory and in the trajectory of Donald J. Trump’s career. At other times, his messages might have greater or smaller effects on his supporters. Moreover, it is not clear whether our findings for Donald J. Trump and his supporters would generalize to other political leaders and their supporters, given the literature suggesting that Trump and his followers have a unique relationship [11, 12].

Future research should deploy these sorts of messages in realistic settings, where actual vaccine behaviors can be observed. Nonetheless, it will be important to use research designs that support causal inference, as does our experimental vignette study.

## Implications

Consistent with other work, we found that political identity is an important driver of behavioral health interventions, and that these intentions can be modified by communication interventions [6–8]. Our opportunistic exploratory study suggests that a political speaker’s endorsement of the COVID-19 vaccine may increase uptake among those who identify with that speaker. An expert’s factually accurate message may not. To counter vaccine hesitancy, future research and practice can exploit communication strategies that are most effective for specific segments of the population.
